# Expression of MMP-14 and CD147 in Gingival Tissue of Patients With and Without Diabetes Mellitus Type II

**DOI:** 10.3390/diagnostics15050609

**Published:** 2025-03-03

**Authors:** Ionut Catalin Botezatu, Maria-Alexandra Martu, Laura Stoica, Ana Emanuela Botez, Pavel Onofrei, Cristina Daniela Dimitriu, Bogdan Vasile Grecu, Ionut Daniel Gafincu Grigoriu, Oana Ciurcanu, Carmen Solcan, Anca Ileana Sin, Elena-Carmen Cotrutz

**Affiliations:** 1 Department of Cell and Molecular Biology, “Grigore T. Popa” University of Medicine and Pharmacy, 16 Universității Street, 700115 Iași, Romania; ionut-catalin.botezatu@umfiasi.ro (I.C.B.); laura.stoica@umfiasi.ro (L.S.); ana-emanuela.botez@umfiasi.ro (A.E.B.); onofrei.pavel@umfiasi.ro (P.O.); vasile-bogdan.grecu@umfiasi.ro (B.V.G.); daniel.gafincu-grigoriu@umfiasi.ro (I.D.G.G.); anca.sin@umfiasi.ro (A.I.S.); elena-carmen.cotrutz@umfiasi.ro (E.-C.C.); 2 Department of Periodontology, Faculty of Dental Medicine, “Grigore T. Popa” University of Medicine and Pharmacy, 16 Universității Street, 700115 Iași, Romania; 3 Department of Biochemistry, “Grigore T. Popa” University of Medicine and Pharmacy, 16 Universității Street, 700115 Iași, Romania; daniela.dimitriu@umfiasi.ro; 4 Department of Dento-Alveolar Surgery, “Grigore T. Popa” University of Medicine and Pharmacy, 16 Universității Street, 700115 Iași, Romania; oana.ciurcanu@umfiasi.ro; 5 Department of Cell and Molecular Biology, University of Agricultural Science and Veterinary Medicine “Ion Ionescu de la Brad”, 700490 Iași, Romania; carmen.solcan@guest.umfiasi.ro

**Keywords:** periodontal disease, diabetes mellitus type 2, EMMPRIN, CD147, MMP-14

## Abstract

**Background:** Diabetes mellitus (DM) is a major risk factor for the development of periodontal disease and aggravates the severity of periodontal conditions. Matrix metalloproteinases (MMPs) are known to degrade periodontal ligament attachment and bone matrix proteins. Increased expression of CD147 is associated with increased synthesis of several MMPs, being a modulator of MMP expression, including that of MMP-14. The purpose of this study was to quantify and compare the expressions of MMP-14 and CD147 in gingival tissues of patients with and without type 2 diabetes mellitus. **Material and Methods:** In this histological study, we included 33 subjects with periodontal disease: 16 patients with type 2 DM (test group) and 17 systemically healthy patients (control group). Tissue fragments were processed using an immunohistochemistry technique to determine immunoreactivity (IR) intensity of MMP-14 and CD147. **Results:** In the group of diabetes patients with periodontitis, 56.2% showed weak positive expressions (+), while 43.8% had intensely positive expressions (+++) of MMP-14. Statistically significant differences between test and control groups (*p* = 0.004, *p* = 0.883, and *p* = 0.002) were found for the membranous IR intensity of MMP-14. In the group of diabetes patients with periodontitis, 56.2% had moderate positive expressions (++) of CD 147, while 43.8% showed intensely positive expressions (+++). Statistically significant differences between the test and control groups were found (*p* = 0.001, *p* = 0.002, and *p* = 0.003) for the membranous IR intensity of CD147. **Conclusions:** The significantly higher membranous IR intensity for MMP-14 and CD 147 demonstrates the role of these biomarkers in the development of periodontal pathology in diabetes patients. It can be assumed that MMP-14 and CD147 could be further investigated as potential predictive biomarkers.

## 1. Introduction

Periodontal disease (PD) is a highly prevalent chronic disease that presents a significant prevalence worldwide. A large percentage of the population, especially after a certain age, becomes susceptible to this pathology [1,2]. Diabetes mellitus is one of the most common comorbidities, and it also increases the risk of periodontal pathology due to the increase in inflammation at the level of the periodontium. Epidemiological studies show that diabetes increases the prevalence and severity of periodontal disease three-fold. At the same time, periodontitis may influence diabetes control. Periodontitis and diabetes are both diseases that impact each other’s evolution and treatment outcomes [1,2,3].

In the last three decades, the major goal of periodontal research has been to identify the molecular mechanisms and to find early predictive and prognostic biomarkers for PD. In chronic periodontitis, the balance between the degradation and synthesis of components from the extracellular matrix influences periodontal attachment levels, and the alveolar bone matrix concentration is affected as well [4,5].

Matrix metalloproteinases (MMPs) are involved in most biological processes. MMPs are a family of Zn-dependent endopeptidases responsible for degrading ECM proteins such as collagen and elastin, essential for tissue remodeling under physiological and pathological conditions [6,7]. Matrix metalloproteinases (MMPs) are known to degrade periodontal ligamental attachment and bone matrix proteins. Among these, MMP-14, anchored to the cell surface, is thought to play a major role in controlling proteolytic events within the pericellular microenvironment. In addition to its role in collagen remodeling, MMP-14 can activate pro-MMP-2 and pro-MMP-13, which may amplify proteolytic potential [8].

CD147 (EMMPRIN or BASIGIN), a transmembrane glycoprotein and a member of the immunoglobulins superfamily, is an adhesive molecule identified on different cell types implicated in fibrosis processes or cell lysis. It contributes to extracellular matrix remodeling either directly or indirectly by targeting CD14 partners. In cancer cells, there is a correlation between overexpression of CD147 and different proteinase expressions, such as MMP-1, -2, -3, -9, and -14 [9]. The expression of EMMPRIN has been directly identified by some authors as a potential biomarker of tumor progression and inflammation, due to its involvement in the induction of proteinases that cause the degradation of the extracellular matrix, tumor invasion and angiogenesis [10,11,12]. CD147 stimulates the production of several matrix metalloproteinases; however, few studies have analyzed the link with periodontal disease evolution [13].

Recently, MMP-14 (MT1-MMP) was reported to be up-regulated in some types of cancer and to promote cancer cell invasion and metastasis. For example, MMP-14 expression is overexposed in different types of tumors like ovarian cancer, gastric, renal or colorectal cancer and is correlated with a bad prognosis. MMP-14 plays a major role in pericellular collagenolysis in vitro and in vivo [13,14,15,16]. Epithelial MMP-14 deficiency can increase the severity of idiopathic pulmonary fibrosis, which reveals a possible anti-fibrotic mechanism [17]. It degrades the extracellular matrix components such as elastin and its precursor tropoelastin, fibronectin and collagen. MMP-14 can also activate other metalloproteinases, like MMP-2 and MMP-13 [18,19,20].

In view of these aspects, the present study evaluated EMMPRIN immunohistochemical expression correlated with MMP14 expression in the periodontal tissue of patients with or without diabetes in order to highlight possible interrelationships between these two molecules and to identify a possible involvement in the molecular pattern regarding the evolution of periodontitis.

## 2. Materials and Methods

This study was conducted in strict adherence to the ethical principles outlined in the Declaration of Helsinki. Prior to participation, informed consent was obtained from all individuals included in the study.

The research was reviewed and approved by the Research Ethics Committee of the University of Medicine and Pharmacy “Grigore T. Popa” Iași, under approval number 359 on 20 November 2023. After receiving ethical approval, participants underwent thorough clinical and paraclinical evaluations.

The study included patients with periodontal disease (stage 2 or 3) aged 18 years or older who required at least one tooth extraction due to periodontal causes. For participants in the type 2 diabetes group, eligibility criteria included a confirmed diagnosis of type 2 diabetes for a minimum of three years (in accordance with the guidelines of the American Diabetes Association) and glycated hemoglobin levels ranging from 6.5% to 11%.

Exclusion criteria were as follows: recent use of antibiotics or anti-inflammatory medications within the last three months, any periodontal or orthodontic procedures within the previous six months, stage 1 and 4 periodontal disease, a body mass index (BMI) greater than 30, psychiatric disorders, pregnancy or breastfeeding, smoking, and systemic conditions that could affect immune function or bone metabolism (e.g., osteoporosis, rheumatoid arthritis, endocrine disorders), except for type 2 diabetes.

In the present study, we used periodontal tissue fragments collected by incisional biopsy with a nr. 15 scalpel blade.

After applying the inclusion and exclusion criteria, we divided the patients in two groups: diabetes mellitus type 2 group (DM + PD): 16 patients with periodontal disease; diabetes mellitus type II and control group (PD): 17 patients with periodontal disease and no diabetes.

The sections were displayed on electrostatically charged slides and examined with the Olympus BX40 microscope (Hachioji, Tokyo) with the Olympus E330 (Hachioji, Tokyo) camera attached, performed within the Department of Cellular and Molecular Biology, at “Grigore T. Popa” University of Medicine and Pharmacy”, Iasi.

Some of the harvested tissue fragments were processed using the paraffin embedding technique and classical hematoxylin-eosin staining in order to achieve a histopathological diagnosis, and other fragments were processed by a standard immunohistochemical technique using anti-MMP-14 antibodies (dilution 1:50, Santa Cruz Biotechnology, Inc., Santa Cruz, CA, USA), anti-EMMPRIN (BSG) Mouse Monoclonal Antibody (in 1/250 µL dilution) and the NovoLink TMMax Polymer Detection System kit produced by Leica Biosystems Newcastle Ltd. Balliol Business ParkWest Benton Lane (Newcastle upon Tyne, UK). Parameters of MMP-14 and CD147 were as follows: temperature 4 °C for the primary antibody in the refrigerator overnight; detection system: DAB.

## 3. Results

Table 1 highlights the demographic characteristics of the analyzed study groups, the cause of dental extraction and the baseline HbA1c value.

**Figure 1:**
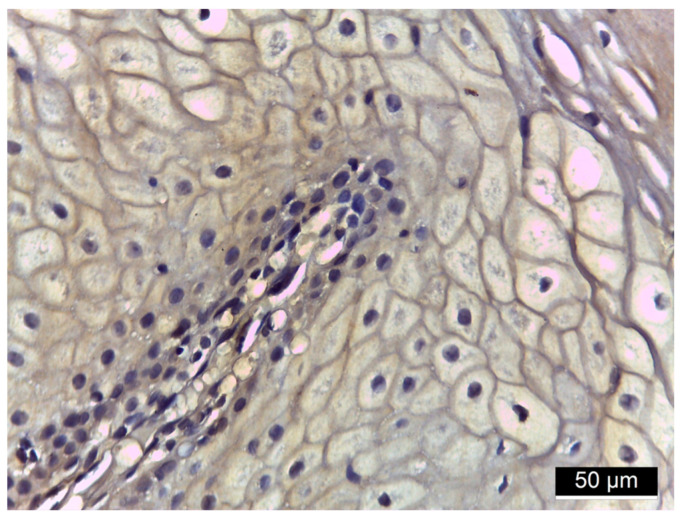
Periodontal tissue fragment from a diabetes mellitus patient, 40× magnification; intensely positive (+++) MMP-14 IR at the cell membrane level in the basal and parabasal layers.

**Figure 2:**
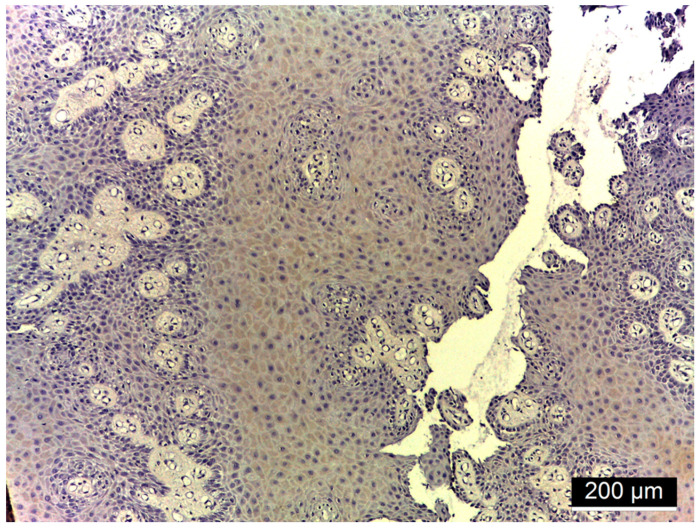
Fragment of periodontal tissue at the level of the gingival sulcus from a diabetes mellitus patient, 20× magnification; diffuse weakly positive (+) MMP-14 IR predominantly in the basal and spinous layers.

**Figure 3:**
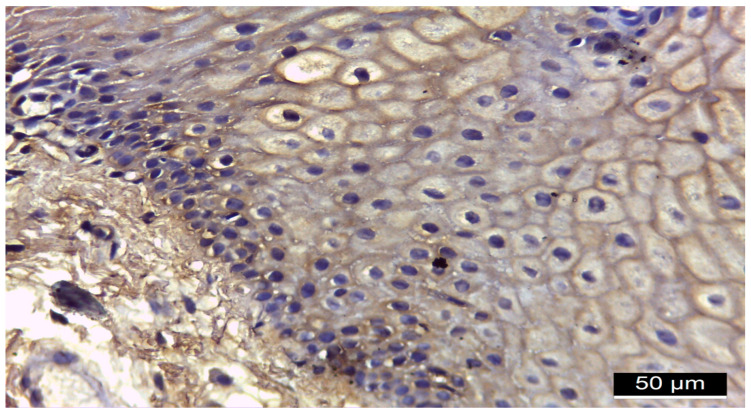
Fragment of periodontal tissue from a diabetes mellitus patient, 20× magnification, intensely positive (+++) CD147 IR at the cell membrane level.

**Figure 4:**
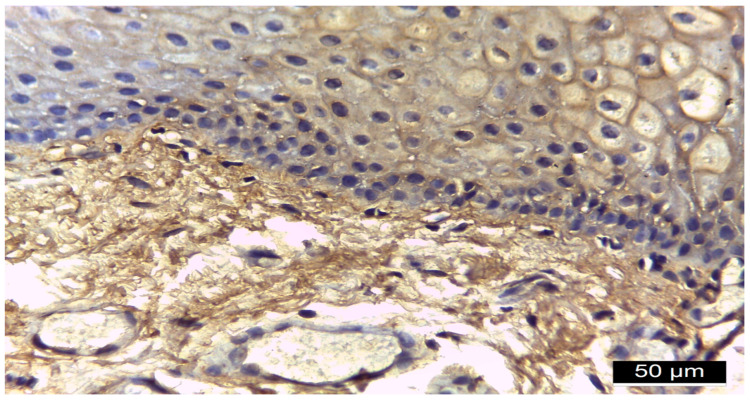
Fragment of periodontal tissue from a diabetes mellitus patient, 20× magnification, moderately positive (++) CD147 IR at the membrane level.

**Figure 5:**
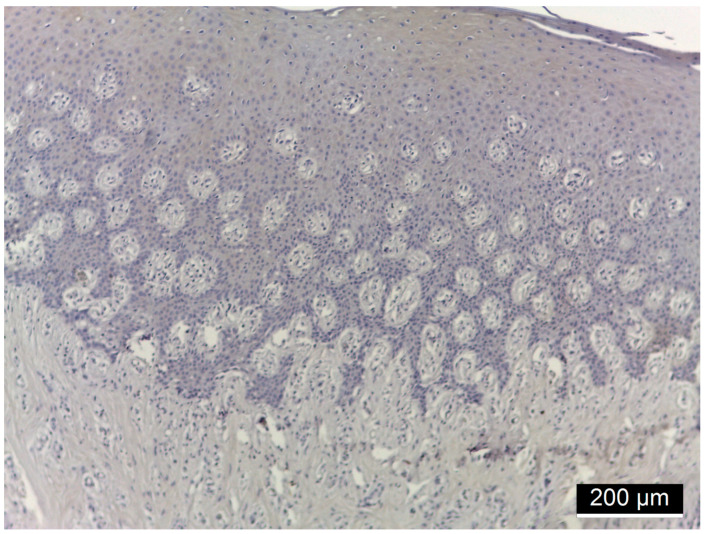
Periodontal tissue fragment from a control patient, 10× magnification; weak (+) MMP-14 IR at the cell membrane level in the parabasal and intermediate layer.

**Figure 6:**
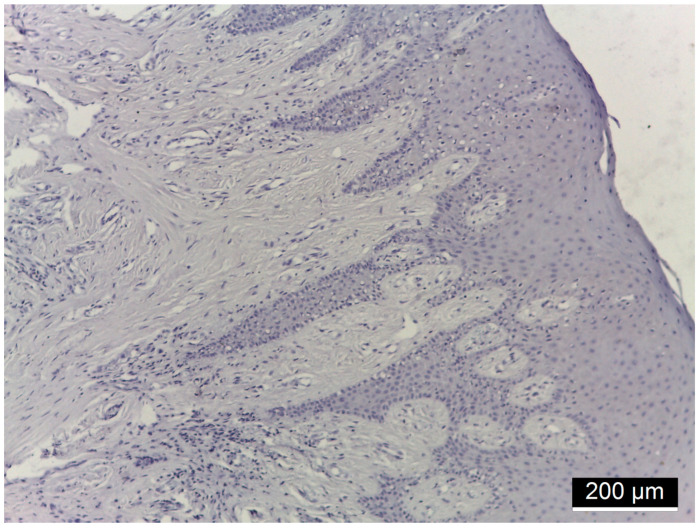
Fragment of periodontal tissue from a control patient, 20× magnification; negative (−) CD147 IR at the cell membrane level.

Figure 1, Figure 2, Figure 3 and Figure 4 illustrate histological findings in diabetes mellitus patients, while Figure 5 and Figure 6 are taken from samples of periodontitis and systemically healthy individuals. In the MMP-14-labeled sample, examination of the periodontal tissue fragments from a diabetes mellitus patient revealed intensely positive immunoreactivity (+++) at the cell membrane level in the basal and parabasal layers membrane (Figure 1). A fragment of the gingival sulcus from a diabetes mellitus patient showed diffuse weakly positive (+) MMP-14 IR predominantly in the basal and spinous layers (Figure 2). In the CD147-labeled sample, examination of periodontal tissue fragments from a diabetes mellitus patient revealed intensely positive (+++) CD147 IR at the cell membrane level (Figure 3). A fragment of periodontal tissue from a diabetes mellitus patient showed moderately positive (++) CD147 IR at the membrane level (Figure 4). In the MMP-14-labeled sample from the control patient, the examination of a periodontal tissue fragment revealed weak (+) MMP-14 IR at the cell membrane level in parabasal and intermediate layers (Figure 5). In the CD 147-labeled sample from the control patient, the examination of a periodontal tissue revealed negative (−) CD147 IR at the cell membrane level (Figure 6).

The membranous expression of MMP-14 varied between the study and test groups. In the group of diabetes patients with periodontitis, 56.2% (9 out of 16 cases) showed weak positive expression (+), while 43.8% (7 out of 16) had intensely positive expression (+++). In contrast, 41.2% (7 out of 17) of the control group showed negative expression (−) and 58.8% (10 out of 17) showed weak positive expression (+). The chi-square test for membranous intensity indicated statistically significant differences between groups (*p* = 0.004, *p* = 0.883, and *p* = 0.002). Cytoplasmic and nuclear MMP-14 expressions were negative in all samples, with no detectable immunoreactivity and no significant differences between the groups (Table 2 and Table 3).

The membranous expression of CD 147 varied between the study and test groups. In the group of diabetes patients with periodontitis, 56.2% (9 out of 16 cases) had moderate positive expression (++), while 43.8% (7 out of 16) showed intensely positive expression (+++). The control group showed 41.2% (7 out of 17) negative expression (−) and 58.8% (10 out of 17) showed weak positive expression (+). The chi-square test for membranous intensity indicated statistically significant differences between groups (*p* = 0.001, *p* = 0.002, and *p* = 0.003). Cytoplasmic and nuclear CD 147 expressions were negative in all samples, with no detectable immunoreactivity and no significant differences between the groups (Table 4 and Table 5).

## 4. Discussion

Several systemic diseases and conditions such as autoimmune diseases, cardiovascular disease, chronic kidney disease, osteoporosis, cancer, depression, and diabetes type 1 and 2 are common aggravating factors for periodontitis that also increase the risk of periodontal pathology due to the elevation of inflammation at the level of the periodontium [21,22,23,24,25].

Diabetes mellitus is characterized by elevated blood sugar levels resulting from disruptions in insulin function, secretion, or both. This condition causes a range of molecular and cellular disruptions, including oxidative stress, an increase in inflammatory responses, and changes to blood vessels [26,27,28,29]. High glucose levels are particularly linked to diabetic complications through the accelerated formation of advanced glycation end products (AGEs). Furthermore, elevated blood sugar is associated with increased concentrations of various inflammatory substances such as interferon-gamma (IFN-γ), tumor necrosis factor-alpha (TNF-α), C-reactive protein (CRP), interleukins (ILs), and matrix metalloproteinases (MMPs) [30,31,32,33,34].

Diabetes can give rise to a variety of complications, one of which is periodontitis, a significant condition alongside issues like cardiovascular disease, kidney damage, retinopathy, neuropathy, and peripheral vascular disorders [35,36,37,38,39,40]. Periodontitis is a chronic inflammatory condition that affects the tissues supporting the teeth, typically caused by bacterial infections. In its more advanced stages, it leads to the breakdown of connective tissue and bone destruction, which may ultimately result in tooth loss in adults [41,42]. A key factor in this process is the bacterial component lipopolysaccharide (LPS), which triggers the activation of macrophages. These macrophages release several pro-inflammatory molecules, including interleukin-1 (IL-1), tumor necrosis factor-alpha (TNF-α), and prostaglandin E2 (PGE2) [43,44,45,46]. Bacterial toxins also activate T lymphocytes, which produce cytokines that promote inflammation and tissue degradation. These cytokines are crucial in the destruction of periodontal tissues through matrix metalloproteinases (MMPs) [47,48,49,50]. In addition, reactive oxygen species (ROS) can stimulate enzymes that break down collagen and increase collagenase activity in inflamed gingival tissues. Elevated levels of C-reactive protein (CRP), a marker of systemic inflammation, are often seen in individuals with periodontal disease, further linking inflammation to the progression of the condition [51,52].

Several studies have emphasized that immune system dysfunction, reduced ability to fight infections, and heightened vulnerability to bacterial attacks are key factors in the onset of periodontal disease in individuals with diabetes. Evidence continues to mount, indicating that the inflammatory response triggered by pathogens plays a critical role in the development of periodontal disease in diabetic patients. In particular, those with both diabetes and periodontitis demonstrate increased production of inflammatory markers in their gum tissues compared to individuals without diabetes [53,54,55,56,57,58,59].

The pathogenesis of both periodontitis and diabetes appears to involve similar immune and inflammatory mechanisms, affecting both local and systemic responses. Despite extensive clinical evidence showing the negative impact of diabetes on periodontal health, there remains a lack of comprehensive data regarding the cascade of immune-inflammatory mediators at the sites of chronic periodontitis in people with diabetes. The simultaneous occurrence of these two conditions suggests that shared mechanisms may drive the development of both [60,61]. It is believed that the inflammation and immune dysregulation caused by diabetes could promote the onset of periodontitis and contribute to the accumulation of dental plaque biofilm. However, the precise mechanisms linking diabetes to periodontitis are still not fully understood. The roles of certain cytokines and molecules, such as IL-4, TNF-γ, and TIMP-2, in driving the progression of periodontal disease and alveolar bone loss in individuals with diabetes, have not been completely clarified [62,63].

In our personal research, the assessment of MMP-14 immunoreactivity revealed statistically significant differences in the intensity of membranous expression between diabetes patients and non-diabetes patients with periodontal pathology. The chi-square test indicated significant *p*-values (*p* = 0.001, *p* = 0.002, *p* = 0.003), confirming a notable variation in MMP-14 membranous expression between the test and the control groups. In contrast, the differences were absent between cytoplasmic and nuclear expressions of MMP-14, with all cases being negative (*p* = 1.000). This finding suggests that MMP-14 is active exclusively at the membrane level and may play a specific role in the pathological mechanisms involved in the development of periodontitis in diabetes patients. Our results support the existing data from the literature, which indicate a primarily membranous role of MMP-14 in extracellular matrix degradation processes and the activation of other matrix metalloproteinases [64].

A rat model study was used to analyze the involvement of inflammatory markers in periodontal destruction associated with diabetes, periodontitis (PD), and the combination of these two conditions. The study found that CRP, MMP-2, MMP-14, TIMP-2, and IFN-γ contribute to the inflammatory processes driving periodontal damage in diabetes. The expression of these markers was significantly higher (*p* < 0.05) when periodontitis developed alongside preexisting diabetes compared to cases where diabetes occurred in subjects with existing periodontitis [64]. A study by Ryu assessed how diabetes mellitus (DM) influences the expression of MMP-3 (stromelysin) and MMP-14 (membrane type) in the gingival tissues of patients with type 2 diabetes and healthy adults with chronic periodontitis. The study found that the expression levels of MMP-3 and MMP-14 were comparable in both healthy and inflamed gingival tissues from individuals without systemic health issues. However, in patients with chronic periodontitis associated with type 2 diabetes, the levels of both MMP-3 and MMP-14 were significantly higher than in healthy or non-diabetic inflamed gingival tissues. Additionally, a correlation between MMP-3 and MMP-14 expression was observed, suggesting that MMP-3 might influence MMP-14 activity in the progression of periodontal disease linked to diabetes. These findings suggest that MMP-3 and MMP-14 could be involved in the advancement of periodontal inflammation in diabetic patients, possibly through interactions with other factors such as advanced glycation end products (AGEs), plasmin, and other MMPs. Consequently, the expression of MMP-3 and MMP-14 may be valuable as markers for periodontal inflammation in individuals with type 2 diabetes [65]. In another study, the expression of PGE2, MMP-14, and TIMP-1 was evaluated in the gingival tissues of individuals with type 2 diabetes and healthy adults with chronic periodontitis, particularly those experiencing alveolar bone resorption. Participants were classified into three groups: Group 1, consisting of clinically healthy gingiva with no signs of bleeding, bone loss, or periodontal pockets; Group 2, which included patients with chronic periodontitis and alveolar bone resorption; and Group 3, composed of patients with chronic periodontitis and alveolar bone resorption associated with type 2 diabetes. The results indicated significantly higher levels of MMP-14, PGE-2 and TIMP-1 in Group 3 when compared to those in both Group 1 and Group 2. Moreover, the study found that MMP-14 and TIMP-1 expression levels were elevated in inflamed gingival tissue, suggesting that PGE2 and MMP-14 may contribute to the process of alveolar bone resorption and the progression of periodontal inflammation associated with type 2 diabetes [66]. A study by Kim in 2011 examined the expression levels of C-reactive protein (CRP), matrix metalloproteinase-14 (MMP-14), and the tissue inhibitor of metalloproteinases-2 (TIMP-2) in gingival tissues from individuals with chronic periodontitis, characterized by inflammation and alveolar bone resorption, both with and without type 2 diabetes mellitus (DM). The results suggested that CRP, MMP-14, and TIMP-2 could act as key inflammatory markers in gingival tissues undergoing periodontal inflammation. These findings imply that CRP, MMP-14, and TIMP-2 may play a contributory role in the progression of periodontal inflammation associated with type 2 diabetes [67].

Extracellular matrix metalloproteinase inducer (EMMPRIN, or CD147) is a highly versatile transmembrane glycoprotein that plays a critical role in regulating cellular behavior and tissue remodeling processes. In addition to its pivotal roles in metabolic regulation and angiogenesis, EMMPRIN is involved in the pathogenesis of numerous diseases characterized by abnormal tissue remodeling and altered cellular interactions [10]. CD147 (EMMPRIN) plays a crucial role in stimulating the expression and activation of various matrix metalloproteinases (MMPs), including MMP-14, which are involved in extracellular matrix (ECM) remodeling as well as tissue degradation. In our study, we found statistically significant differences in the membranous expression intensity of CD 147 between the test and control groups. The chi-square test indicated significant *p*-values (*p* = 0.001, *p* = 0.002, *p* = 0.003), confirming high variation in CD147 membranous expression between the diabetes patients and non-diabetes patients with periodontitis. However, cytoplasmic and nuclear expressions of CD147 showed no differences between test and control groups, all cases being negative (*p* = 1.000). This result suggests that CD147 is predominantly active at the membrane level and may play a specific role in the pathological mechanisms involved in the development of periodontal disease in patients suffering from diabetes.

One of the most profound roles of EMMPRIN is its involvement in the regulation of aerobic glycolysis, a process that is particularly important for rapidly proliferating cells such as those in tumors or tissues undergoing extensive repair. In addition to its metabolic roles, EMMPRIN influences the expression and activity of glucose transporter GLUT1, which is responsible for the uptake of glucose into cells. These actions highlight EMMPRIN’s ability to fine-tune glucose uptake in response to cellular needs, further supporting its role in metabolic homeostasis and disease progression [68].

Beyond its metabolic and glucose-regulating functions, EMMPRIN is a potent inducer of matrix metalloproteinases. By activating MMPs, EMMPRIN facilitates the remodeling of the extracellular matrix, which is necessary for processes such as endothelial cell migration, tissue repair, and angiogenesis. Angiogenesis, the formation of new blood vessels from pre-existing ones, is a critical process in both normal tissue regeneration and in pathological conditions like cancer. EMMPRIN’s ability to promote the degradation of ECM components and support endothelial migration makes it a key player in angiogenesis. Moreover, EMMPRIN enhances the expression of vascular endothelial growth factor (VEGF), a major pro-angiogenic factor that drives blood vessel formation [69].

The diverse biological functions of EMMPRIN are largely governed by its glycosylation at three specific N-linked sites, which modulate its interactions with other proteins and its overall functional activity. Aberrant glycosylation of EMMPRIN can lead to dysregulated cellular behavior and contribute to disease progression, particularly in cancers and conditions associated with abnormal angiogenesis. Although EMMPRIN’s role in cancer and angiogenesis is well documented, its involvement in diabetic complications, particularly those involving abnormal angiogenesis like diabetic retinopathy, remains an area of active investigation [70].

In oral squamous cell carcinoma (OSCC), CD147 is significantly overexpressed in both tumor cells and the surrounding normal cells within the tumor microenvironment. Its expression and activity show a gradual increase during the progression from healthy oral mucosa to inflammation, through hyperplastic and dysplastic changes, and eventually to OSCC development [71].

EMMPRIN and Caveolin-1 (Cav-1) have been identified as significant contributors to the pathogenesis of periodontal disease, yet there is limited understanding of how they interact and influence periodontal inflammation. A study that included 30 participants revealed a significant increase in EMMPRIN levels and a decrease in Cav-1 levels as periodontal disease advanced (*p* < 0.01). After periodontal therapy, EMMPRIN levels decreased, while Cav-1 levels increased significantly (*p* < 0.01). These results suggest a positive correlation between EMMPRIN expression and the extent of periodontal inflammation, while Cav-1 expression shows an inverse relationship. EMMPRIN appears to facilitate tissue destruction in periodontal disease, whereas Cav-1 helps reduce inflammation. This highlights the complex interaction between EMMPRIN and Cav-1 in periodontal disease progression and underscores the potential of periodontal therapy to restore a balanced expression of these markers [72].

Moreover, it appears that EMMPRIN glycosylation is integral to the regulation of MMP-2 and MMP-9 production through its mediation of the interactions between human oral endothelial cells (HIOECs) and human gingival fibroblasts (HGFs). The inhibition of EMMPRIN glycosylation leads to a significant reduction in the activation of MMP-2 and MMP-9, which in turn suppresses the degradation of the extracellular matrix (ECM) in the HIOEC/HGF co-culture system. These findings suggest that EMMPRIN glycosylation plays a pivotal role in regulating the host’s immune-inflammatory response in periodontitis by controlling MMP activity. This understanding opens potential avenues for targeted therapeutic approaches that could reduce tissue destruction and inflammation in periodontal disease, ultimately contributing to more effective treatments [73].

Immunohistochemical analysis revealed significant differences in the immunopositivity for MMP-7, EMMPRIN, and CypA, with the highest expression levels observed in chronic periodontitis samples, followed by chronic gingivitis and healthy gingiva samples. Notably, the expression of CypA and MMP-7 was more prominent in areas with dense inflammatory infiltrates, which were primarily present in periodontitis cases. A robust positive correlation was found between CypA and MMP-7 expression, while a moderate positive correlation was observed between CypA and EMMPRIN. Additionally, probing depth showed significant positive correlations with MMP-7, EMMPRIN, and CypA. This study suggests that MMP-7, EMMPRIN, and CypA are closely linked to the development and progression of periodontal disease, with their expression levels reflecting the degree of periodontal tissue destruction. The significant correlations with probing depth further highlight their potential as reliable markers of periodontal inflammation [74].

The expression of EMMPRIN was detected in various cell types within the periodontal tissues, including gingival epithelial cells, inflammatory cells, endothelial cells, and fibroblast-like cells. Double immunofluorescence analysis confirmed the co-localization of EMMPRIN and caveolin-1 in gingival epithelium, endothelial cells, and fibroblast-like cells, suggesting an interaction between these two molecules. In the chronic periodontitis group, there was a significant increase in high-glycoform EMMPRIN (HG-EMMPRIN) and active MMP-1 compared to healthy controls (*p* < 0.05). Additionally, a positive correlation was observed between HG-EMMPRIN levels and both active MMP-1 and proMMP-1 protein levels. Thus, the co-localization of EMMPRIN and caveolin-1 in periodontal tissues may play a crucial role in the pathogenesis of periodontal disease. Elevated levels of HG-EMMPRIN are associated with the increased production of active MMP-1 and proMMP-1, which are key mediators of tissue breakdown in chronic periodontitis [75].

Another interesting study observed that in the particular case of an inflammatory lesion, blood vessel morphology displayed modifications according to the progression of gingival lesions, culminating in the seeming appearance of intussusception in severe inflammation [76].

The limitations of our study include the reduced number of patients, limited diagnostic measures applied, absence of other paraclinical investigations and lack of follow-up. Also, considering this is a histological study, the conclusions should be interpreted with caution and further studies are necessary for improved clinical correlations.

These observations provide insights into the molecular mechanisms driving periodontal destruction and highlight potential therapeutic targets for regulating these pathways in periodontal disease.

In the context of diabetes, EMMPRIN may influence the development of pathological blood vessels and contribute to the progression of microvascular complications. Further research into the precise role of EMMPRIN in diabetic angiogenesis could provide valuable insights for the development of targeted therapies aimed at managing or preventing vascular complications in diabetic patients, such as retinopathy, nephropathy, and impaired wound healing [77,78,79,80,81,82,83].

Understanding the molecular mechanisms through which MMP-14 and EMMPRIN regulates pathological processes could have significant implications for the design of novel therapeutic strategies. By modulating its expression or activity, it may be possible to influence the progression of diseases involving abnormal tissue remodeling, including diseases such as diabetes and periodontitis.

## 5. Conclusions

The notable difference in membranous IR intensity between test and control groups suggests that both MMP-14 and CD 147 may play a role in the development of periodontal pathology for diabetes patients.

The absence of cytoplasmic and nuclear expression of MMP-14 and CD 147 across all patients suggests that these markers are not active in these particular cellular compartments for both diabetes and non-diabetes patients with periodontitis.

From the limited samples obtained, we can assume that MMP-14, and CD147 are involved in the progression of periodontal disease associated with type 2 DM and could be further investigated as potential predictive biomarkers.

## Figures and Tables

**Table 1:** Descriptive data for the study participants.

Parameters	All Cases (*n* = 33)	Control Group (*n* = 17)	Diabetes Mellitus Group (*n* = 16)
Age (years), mean	55.4	52.5	57.8
(min–max)	(28–75)	(28–72)	(29–75)
Gender			
Male *n* (%)	16 (44.4%)	7 (41.18%)	9 (56.2%)
Female *n* (%)	17 (47.2%)	10 (58.8%)	7 (43.7%)
Area			
Urban	22 (61.1%)	12 (70.5%)	10 (62.5%)
Rural	11 (33.3%)	5 (29.4%)	6 (37.5%)
HbA1c (%), mean	4.2	5	7.9
(min–max)	(4.2–9.3)	(4.3–5.7)	(6.8–9.3)
Periodontal disease stage			
2	13 (39.39%)	7 (41.1%)	6 (37.5%)
3	20 (60.6%)	10 (58.8%)	10 (62.5%)

HbA1c—glycated hemoglobin.

**Table 2:** Distribution of patients (test; control) related to MMP-14 immunoreactivity intensity.

Patients Number	Diagnostic	Immunoreactivity Intensity MMP-14		
		Membranous	Cytoplasmatic	Nuclear
9	Diabetes mellitus + Periodontitis	+	−	−
7	Diabetes mellitus + Periodontitis	+++	−	−
10	Periodontitis (control)	+	−	−
7	Periodontitis (control)	−	−	−

**Table 3:** Comparison of immunoreactivity intensity MMP-14 (test vs. control).

Location	IR Intensity	DM + PD *n* = 16	PD *n* = 17	Chi Square Test *p*
	**MMP-14**			
**Membranous**	(−) (+) (++) (+++)	9 (56.2%) 7 (43.8%)	7 (41.2%) 10 (58.8%)	0.004 0.883 nc 0.002
**Cytoplasmic**	(−) (+) (++) (+++)	16 (100%)	17 (100%)	1.000 nc nc nc
**Nuclear**	(−) (+) (++) (+++)	16 (100%)	17 (100%)	1.000 nc nc nc

nc—not computed.

**Table 4:** Distribution of patients (test; control) related to CD147 immunoreactivity intensity.

Patients Number	Diagnostic	Immunoreactivity Intensity CD147		
		Membranous	Cytoplasmatic	Nuclear
9	Diabetes mellitus + Periodontitis	++	−	−
7	Diabetes mellitus + Periodontitis	+++	−	−
10	Periodontitis (control)	+	−	−
7	Periodontitis (control)	−	−	−

**Table 5:** Comparison of immunoreactivity intensity CD-147 (test vs. control).

Location	IR Intensity	DM + PD *n* = 16	PD *n* = 17	Chi Square Test *p*
	**CD 147**			
**Membranous**	(−) (+) (++) (+++)	9 (56.2%) 7 (43.8%)	7 (41.2%) 10 (58.8%)	0.004 0.001 0.001 0.002
**Cytoplasmic**	(−) (+) (++) (+++)	16 (100%)	17 (100%)	1.000 nc nc nc
**Nuclear**	(−) (+) (++) (+++)	16 (100%)	17 (100%)	1.000 nc nc nc

nc—not computed.

## Data Availability

Data supporting reported results can be provided by the corresponding authors upon reasonable request.
